# Impaired innate immune gene profiling in airway smooth muscle cells from chronic cough patients

**DOI:** 10.1042/BSR20171090

**Published:** 2017-11-15

**Authors:** Christos Rossios, Stelios Pavlidis, David Gibeon, Sharon Mumby, Andrew Durham, Oluwaseun Ojo, Daniel Horowitz, Matt Loza, Fred Baribaud, Navin Rao, Kian Fan Chung, Ian M. Adcock

**Affiliations:** 1 Airway Disease Section, National Heart and Lung Institute, Imperial College London, London; 2 NIHR Respiratory Biomedical Research Unit, Royal Brompton NHS Foundation Trust, London, UK; 3 R&D IT External Innovation, Janssen R&D, High Wycombe, U.K.; 4 Immunologic Therapeutic Area, Janssen R&D, Inc., Spring House, PA, U.S.A.

**Keywords:** airway smooth muscle vcells, Chronic cough, cytokines, innate iimunity, steroids

## Abstract

Chronic cough is associated with airway inflammation and remodelling. Abnormal airway smooth muscle cell (ASMC) function may underlie mechanisms of chronic cough. Our objective was to examine the transcriptome and focused secretome of ASMCs from chronic cough patients and healthy non-cough volunteers. ASMC gene expression profiling was performed at baseline and/or after stimulation with polyinosinic:polycytidylic acid (poly(I:C)) to mimic viral infection. Supernatants were collected for multiplex analysis. Our results showed no significant differentially expressed genes (DEGs, false discovery rate (FDR) <0.05) between chronic cough and healthy non-cough ASMCs at baseline. Poly(I:C) stimulation resulted in 212 DEGs (>1.5 fold-change, FDR <0.05) in ASMCs from chronic cough patients compared with 1674 DEGs in healthy non-cough volunteers. The top up-regulated genes included chemokine (C–X–C motif) ligand (CXCL) 11 (*CXCL11*), *CXCL10*, chemokine (C–C motif) ligand (CCL) 5 (*CCL5*) and interferon-induced protein 44 like (*IFI44L*) corresponding with inflammation and innate immune response pathways. ASMCs from cough subjects had enhanced activation of viral response pathways in response to poly(I:C) compared with healthy non-cough subjects, reduced activation of pathways involved in chronic inflammation and equivalent activation of neuroregulatory genes. The poly(I:C)-induced release of inflammatory mediators, including CXCL8, interleukin (IL)-6 and CXCL1, from ASMCs from cough patients was significantly impaired compared with healthy non-cough subjects. Addition of fluticasone propionate (FP) to poly(I:C)-treated ASMCs resulted in greater gene expression changes in healthy non-cough ASMCs. FP had a differential effect on poly(I:C)-induced mediator release between chronic cough and healthy non-cough volunteers. In conclusion, altered innate immune and inflammatory gene profiles within ASMCs, rather than infiltrating cells or nerves, may drive the cough response following respiratory viral infection.

## Introduction

Chronic cough defined as the cough lasting for more than 8 weeks is a common clinical problem present in a proportion of the population [1,2]. A heightened cough reflex is a common abnormality observed in people with a chronic cough, which can be disabling and impairs quality of life [3,4]. Cough variant asthma and eosinophilic bronchitis are two common causes of chronic cough associated with eosinophilia that respond to anti-inflammatory corticosteroids [5,6]. On the other hand, some forms of chronic cough in which no identifiable cause is found, termed as idiopathic cough, can also be associated with inflammatory and airway wall remodelling features in the airways submucosa including an increase in mast cells, goblet cell hyperplasia, increased blood vessels and airway smooth muscle hypertrophy and hyperplasia [7]. Elevated levels of inflammatory mediators including chemokine (C–X–C motif) ligand (CXCL) 8 (CXCL8), tumour necrosis factor α (TNFα) and myeloperoxidase, lipids such as prostaglandin (PG)D_2_ and PGE_2_, and leucotriene (LT) B4 and neutrophils are present ιn induced sputum of patients with persistent cough [8]. Increased numbers of mast cells and eosinophils as well as elevated histamine levels have been reported ιn bronchoalveolar lavage fluid (BALF) of patients with chronic cough [9]. The mechanisms by which chronic cough occurs are unclear but there is growing evidence that the interaction of inflammatory factors with epithelial nerves in the airways may be important [10].

An abnormality of the airway smooth muscle cell (ASMC) function in chronic cough is suggested by the increased mass seen in patients with chronic cough [7,11], which may result from an increased proliferative rate. In addition, because ASMCs are known to possess the ability to generate inflammatory mediators such as cytokines, chemokines, proteases and growth factors [12], it is possible that these cells may contribute to the inflammatory process that underlines potential neuroinflammatory mechanisms [13]. ASMCs from patients with asthma are in a hyperproliferative phase, overexpress the chemokines chemokine (C–C motif) ligand (CCL) 11 (CCL11) and CXCL8, and particularly in patients with severe asthma, there is a reduced effect of corticosteroids in inhibiting TNFα-induced release of CCL11 and CXCL8 [14,15]. In addition, in asthma there is an increased number of mast cells in ASM bundles that could set the scene for an interaction of the Th2 cytokines, interleukin (IL)-4 and IL-13, released from mast cells to interact with ASMCs [16]. Whether there are related inflammatory or innate immune abnormalities in the ASMCs from patients with chronic cough is not known. Such changes in ASMCs may lead to inflammatory changes around nerve terminals in the airways for the induction of neuroinflammatory interactions.

In this pilot study, we isolated ASMCs from the airways and after culturing, we examined their mRNA and secretome profile to determine whether ASMCs from non-asthmatic chronic cough patients of no known cause were different from those from healthy non-coughing volunteers. Because respiratory viral infections such as the common cold are a common cause of acute cough and because chronic idiopathic cough is very often initiated by the common cold, we analysed ASMCs under stimulation with polyinosinic:polycytidylic acid (poly(I:C)), a viral mimic; in addition, we examined the effect of the corticosteroid, fluticasone propionate (FP), since this is often used as a treatment for chronic cough. Corticosteroids however while efficacious in controlling cough associated with asthma, cough variant asthma and eosinophilic bronchitis, it is not efficacious in non-asthmatic chronic cough [1].

## Methods

### Patients

Patients with chronic cough were recruited from a cough clinic at the Royal Brompton and Harefield (RBH) NHS Trust Hospitals (Table 1). All subjects had reported a dry and persistent cough for at least 8 weeks and had been screened to exclude the diagnosis of other respiratory diseases including asthma. Healthy non-cough volunteers had no smoking history or history of respiratory or cardiac diseases. Chronic cough volunteers were all non-smokers. None of the subjects studied in either group had any evidence for small airway disease/obstruction. The present study was approved by the Ethics Committees of the RBH Hospitals NHS Trust Ethics Committee (11/H0706/4 and 10/H0721/66) and all subjects gave their full informed written consents.

**Table 1:** Demographics of subjects used for the study

	Non-cough volunteers	Cough patients
Age (years)	47.5 ± 4.7	53.5 ± 6.2
Male/female	8/3	2/4
FEV_1_% pred.	91.9 ± 3.9	105.5 ± 5.4
FEV_1_/FVC	77.9 ± 1.3	76.0 ± 3.0
ICS (FP equivalent) (μg/day)	0	50 ± 22.36**

**P=0.002, Mann–Whitney test. No subjects in either group were current or ex-smokers. Abbreviation: ICS, inhaled corticosteroid.

### Fibreoptic bronchoscopy and endobronchial biopsies

Seventeen volunteers (6 cough patients and 11 healthy non-cough volunteers) underwent fibreoptic bronchoscopy. This procedure was performed under sedation according to standard clinical practice at the RBH NHS Trust Hospital. Endobronchial biopsies were collected from the right middle lobe (RML) and were immediately transferred in culture medium containing FBS.

### ASMC isolation and culture

ASMCs were isolated from biopsies and cultured in supplemented Dulbecco’s modified Eagle’s medium (DMEM) as previously described [17]. ASMCs showed a characteristic ‘hill and valley’ morphology and expressed smooth muscle α-actin in more than 95% of the cells. Cells between passages 4 and 6 were used and were FBS deprived for 24 h prior to experiments. During this time they were incubated in Phenol Red-free DMEM supplemented with 4 mM L-glutamine, 20 U/l penicillin, 20 μg/ml streptomycin, 2.5 μg/ml amphotericin B, 1:100 non-essential amino acids and 0.1% BSA. ASMs were stimulated with poly(I:C) (5 μg/ml) for 18 h in the presence or absence of FP (10^−8^ M) added 2 h prior to stimulation.

### ASMC gene analysis

ASMC pellets were used to prepare RNA using the QIA symphony sample prep module (SP) (Qiagen, CA, U.S.A.) and the quality was analysed by LabChip or Bioanalyzer. RNA samples were amplified with Ovation® Pico V2 Automation Kit (Nugen, CA, U.S.A.), fragmented and cDNA labelled using the LabChipGX and NuGEN Encore Biotin Module before analysis on Affymetrix GeneChip® HT HG-U133+ PM microarrays (Affymetrix, CA, U.S.A.). Expression analysis was performed using the OmicSoft Array Studio (v 7.0) (OmicSoft Corporation, NC, U.S.A.) using a general linear model.

### Multiplex assay

Thirty two human cytokines were assayed using the Luminex MAGPIX Analyzer (Austin, TX, U.S.A.) as previously described [18]. The mean fluorescent intensity was analysed using a five-parameter logistic method on XLfit software v.5.3.1.3 (Guildford, Surrey, U.K.).

### Statistical analysis

Affymetrix arrays were normalized using the robust multi-array average (RMA) approach. Quality control (QC) was performed using the median absolute deviation (MAD) residual mean and the relative log expression (RLE) mean to exclude outliers. Arrays that passed QC were used for differential gene expression (DEG) analysis between chronic cough and healthy non-cough subjects at baseline and between treatment groups using a general linear model. Other data are expressed as mean ± S.D. Statistical analysis was calculated by ANOVA for repeated measures. If significant, post tests were performed. Paired comparisons were compared using paired *t* test. Differences were considered significant when *P*<0.05. Statistical analysis was performed using Prism 5 for Windows, v. 5.03 by GraphPad Software, Inc. (La Jolla, CA, U.S.A.).

## Results

### DEGs and proteins in ASMCs at baseline

Although 150 genes were either up- or down-regulated >1.5-fold in cells from cough and non-cough healthy volunteers (Supplementary Table S1), these differences were not significant (*P*<0.05) when analysed by false discovery rate (FDR). Pathway analysis showed the up-regulation of pathways associated with post-translational modifications including histone acetylation and arginine methylation in chronic cough cells.

**Figure 1:**
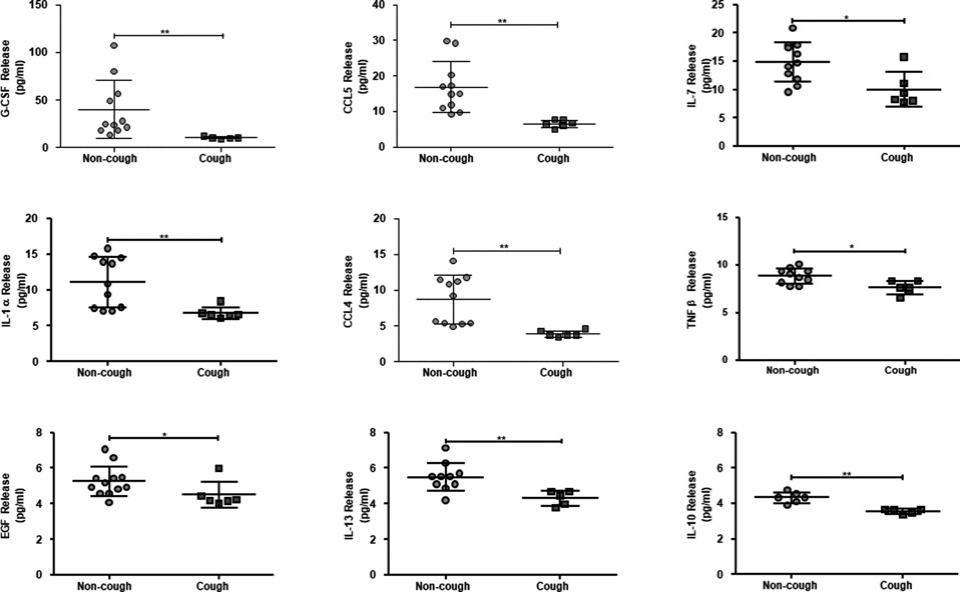
Comparison of baseline secretion of inflammatory mediators from ASMCs from healthy non-cough volunteers and chronic cough patients Unstimulated ASMCs from healthy volunteers (*n*=11) and chronic cough patients (*n*=6) were incubated for 18 h, supernatants were collected and a 32-analyte multiplex bead assay was performed. Data are presented as mean ± S.D.; *P<0.05, **P<0.01.

There was a small but significant (*P*<0.05) reduction in the baseline release of a number of inflammatory and innate immune mediators including TNFβ, IL-1α, IL-7, IL-10, IL-13, CCL4, CCL5, epidermal growth factor (EGF) and granulocyte-colony stimulating factor (G-CSF) in ASMCs from chronic cough patients compared with healthy non-cough volunteers (Figure 1).

### DEGs in poly(I:C)-stimulated ASMCs

Poly(I:C) stimulation of ASMCs regulated similar genes in both chronic cough patients and healthy non-cough volunteers but the response in healthy non-cough volunteers was greater (1674 DEGs: 897 up-regulated, 777 down-regulated) than that seen in ASMCs from chronic cough patients (221 genes up-regulated, 55 down-regulated) when compared with baseline (Supplementary Table S2). Poly(I:C), as expected, up-regulated genes associated with inflammation, innate immunity and viral responses including *CXCL11*, *CCL5* (chemokine (C–C motif) ligand) 5 and *CXCL10*, interferon-induced protein 44 like (IFI44L) in ASMCs from both healthy non-cough and chronic cough volunteers.

The pathway called the effect of alcohol exposure on the brain (alcoholism) is up-regulated to a similar extent by poly(I:C) in cells from cough patients and healthy non-cough volunteers (Table 2). This pathways contains many genes linked to sensory nerve activity or cough including the dopamine D1 receptor, neuropeptide Y, brain derived neurotrophic factor and NMDA-type subunit 1.

**Table 2:** Differential pathway analysis of poly(I:C)-stimulated ASMCs from chronic cough patients compared with cells from healthy non-cough volunteers

Term name	KEGG	Cough		Non-cough	
		Down	Up	Down	Up
Herpes simplex infection	05168	-	2.67E-16	-	5.28E-14
Viral carcinogenesis	05203	-	6.15E-15	-	2.02E-09
Influenza A	05164	-	1.38E-13	-	2.09E-10
Antigen processing and presentation	04612	-	7.04E-10	-	3.96E-06
NF-κB signalling pathway	04064	-	2.45E-09	-	2.81E-10
Measles	05162	-	1.11E-08	-	2.03E-07
Systemic lupus erythematosus	05322	-	6.18E-07	-	4.69E-12
RIG-I-like receptor signalling pathway	04622	-	2.26E-06	-	8.09E-07
TNF signalling pathway	04668	-	4.54E-06	-	1.14E-13
Hepatitis C	05160	-	5.93E-06	-	5.40E-05
Toll-like receptor signalling pathway	04620	-	1.98E-05	-	4.18E-05
Graft-versus-host disease	05332	-	2.26E-04	-	3.07E-03
NOD-like receptor signalling pathway	04621	-	4.55E-04	-	2.84E-04
Epstein–Barr virus infection	05169	-	7.24E-04	-	-
Cytosolic DNA sensing pathway	04623	-	8.66E-04	-	2.66E-05
Alcoholism	05034	-	1.16E-03	-	1.09E-05
Allograft rejection	05330	-	2.16E-03	-	1.79E-03
Legionellosis	05134	-	3.42E-03	-	2.87E-05
Viral myocarditis	05416	-	3.85E-03	-	2.85E-02
Type I diabetes mellitus	04940	-	5.48E-03	-	8.01E-03
Cytokine–cytokine receptor interaction	04060	-	1.55E-02	-	1.06E-07
Autoimmune thyroid disease	05320	-	1.70E-02	-	-
Hepatitis B	05161	-	1.91E-02	-	-
African trypanosomiasis	05143	-	2.20E-02	-	1.35E-03
Chemokine signalling pathway	04062	-	3.02E-02	-	1.96E-03
Apoptosis	04210	-	-	-	1.40E-04
Cell adhesion molecules (CAMs)	04514	-	-	-	4.67E-03
Chagas disease (American trypanosomiasis)	05142	-	-	-	2.72E-02
Hematopoietic cell lineage	04640	-	-	-	8.72E-03
HTLV-I infection	05166	-	-	-	1.63E-02
Inflammatory bowel disease (IBD)	05321	-	-	-	2.07E-02
Jak-STAT signalling pathway	04630	-	-	-	3.71E-03
Leishmaniasis	05140	-	-	-	4.99E-02
Malaria	05144	-	-	-	1.20E-03
Osteoclast differentiation	04380	-	-	-	4.42E-02
Rheumatoid arthritis	05323	-	-	-	2.93E-05
Ribosome	03010	2.15E-07	-	3.92E-10	-
Transcriptional misregulation in cancer	05202	-	-	-	1.11E-04
Tuberculosis	05152	-	-	-	1.93E-03
Valine, leucine and isoleucine degradation	00280	-	-	3.70E-02	-

FDR <0.05. Fold change < –1.5 or >1.5.

Pathways associated with viral infection and immunity were up-regulated in ASMCs from chronic cough patients but not in healthy non-cough volunteers’ ASMCs. In addition, ASMCs from chronic cough patients showed evidence of reduced inflammatory pathway stimulation, such as the TNFα, JAK-STAT and chronic inflammation (rheumatoid arthritis and inflammatory bowel disease) pathways compared with cells from healthy non-cough subjects in response to poly(I:C). Pathway analysis also demonstrated reduced activation of metabolic pathways in response to poly(I:C) stimulation in cells from healthy non-cough volunteers compared with chronic cough patients (Table 2).

**Figure 2:**
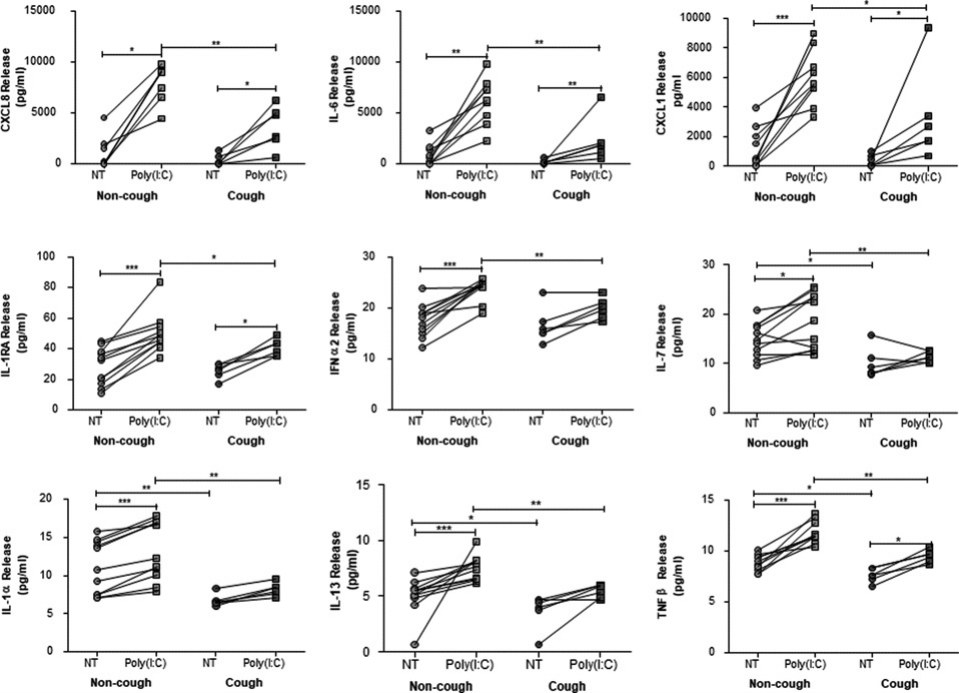
Effect of poly(I:C) on the induction of inflammatory mediators from ASMCs from healthy volunteers and chronic cough patients ASMCs from healthy volunteers (*n*=11) and chronic cough patients (*n*=6) were stimulated for 18 h with 5 μg/ml poly(I:C) or left untreated (NT). Supernatants were collected and the release of 32 different cytokines/chemokines was measured by a multiplex bead assay. Data are represented as mean ± S.D.; *P<0.05, **P<0.01, ***P<0.001.

Poly(I:C) had differential effects on inflammatory and innate immune mediator release in ASMCs from healthy non-cough volunteers and chronic cough patients (Figure 2, Supplementary Table S3). Poly(I:C) significantly induced the release of CX3CL1, CCL11 and EGF in ASMCs from healthy non-cough volunteers only. In contrast, the release of CCL3 and monocyte chemotactic protein 1 (MCP-1) was only induced by poly(I:C) in ASMCs from chronic cough patients. The release of poly(I:C)-induced CXCL8, IL-6, CXCL1, IL-1RA, IFNα2, IL-7, IL-1α, IL-13 and TNFβ was significantly impaired in ASMCs from chronic cough patients compared with that seen in healthy non-cough volunteers (Figure 2, Supplementary Table S3). Overall, there was a reduction in inflammatory gene and protein expression in cells from chronic cough patients compared with healthy non-cough subjects.

### Effect of FP on baseline expression of innate immune and inflammatory genes

Pretreatment of ASMCs from healthy non-cough volunteers with FP resulted in 169 DEGs (118 up-regulated, 51 down-regulated, >1.5-fold change, FDR *P*<0.05) and of 39 DEGs (29 up-regulated, 10 down-regulated, >1.5-fold change, FDR *P*<0.05) in cells from chronic cough patients (Supplementary Table S4). The corticosteroid responsive genes *FKBP5* (FK506 binding protein 5) and *DUSP1* (dual specificity phosphatase 1) were up-regulated by FP and periostin (*POSTN*) and the glucocorticoid receptor (*NR3C1*) were down-regulated by FP to a similar extent in both cough and healthy non-cough volunteers (Supplementary Table S4).

Pathway analysis indicated a significant up-regulation of the systemic lupus erythematosus pathway by FP reflecting Th1-type disease processes in cells from chronic cough patients. No pathways were differentially suppressed in chronic cough patients when compared with healthy non-cough volunteers (Table 3) and FP had no effect on mediator release at baseline.

**Table 3:** Differential pathway analysis of FP-treated ASMCs from chronic cough patients compared with healthy non-cough volunteers

Term name	KEGG	Up-regulated	Down-regulated
Systemic lupus erythematosus	05322	4.62E-10	-
Alcoholism	05034	4.07E-08	-
Viral carcinogenesis	05203	2.51E-07	-

Comparison for DEGs, fold change < –1.5 or >1.5, raw *P*-value <0.05.

### Comparative effects of FP on poly(I:C)-stimulated ASMCs

FP induced more gene expression changes in poly(I:C)-stimulated healthy non-cough cells (490 up-regulated, 699 down-regulated) than in ASMCs from cough patients (49 up-regulated, 119 down-regulated). The top up- and down-regulated genes are shown in Supplementary Table S5 and were similar in each subject group. FP had a differential effect on the expression of a number of genes including a failure to down-regulate *IFI44* and the protein phosphatase *PPM1K* (protein phosphatase, Mg^2+^/Mn^2+^ dependent, 1K) in healthy non-cough ASMCs (Supplementary Table S5).

Pathway analysis highlighted up-regulation by FP of inflammatory, immune and mitochondrial/metabolic pathways in poly(I:C)-stimulated ASMCs from healthy non-cough volunteers compared with chronic cough (Table 4). In contrast, some inflammatory and innate immune/viral signalling pathways were down-regulated by FP in both groups (Table 4). Pathways relating to inflammation and innate immune responses were only down-regulated in ASMCs from healthy non-cough volunteers with no effect seen in cells from chronic cough. In contrast, pathways such as viral carcinogenesis and systemic lupus erythematosus were preferentially down-regulated in cells from chronic cough patients.

**Table 4:** Effect of FP on poly(I:C)-stimulated pathways in ASMCs from chronic cough patients and healthy non-cough volunteers

Term name	KEGG	Cough		Non-cough	
		Up	Down	Up	Down
Viral carcinogenesis	05203	-	6.54E-11	-	-
Systemic lupus erythematosus	05322	-	3.13E-10	-	-
Herpes simplex infection	05168	-	2.88E-07	-	5.57E-10
Influenza A	05164	-	1.83E-06	-	3.50E-05
Alcoholism	05034	-	2.67E-06	-	-
Measles	05162	-	1.45E-03	-	2.01E-02
RIG-I-like receptor signalling pathway	04622	-	2.41E-02	-	1.90E-05
Valine, leucine and isoleucine degradation	00280	-	-	1.53E-05	-
Longevity regulating pathway: mammal	04211	-	-	3.29E-04	-
AMPK signalling pathway	04152	-	-	1.05E-03	-
FoxO signalling pathway	04068	-	-	2.43E-03	-
Insulin resistance	04931	-	-	3.55E-02	-
Antigen processing and presentation	04612	-	-	-	2.24E-06
Cytosolic DNA-sensing pathway	04623	-	-	-	2.44E-05
Cytokine–cytokine receptor interaction	04060	-	-	-	2.54E-04
Toll-like receptor signalling pathway	04620	-	-	-	8.88E-04
Graft-versus-host disease	05332	-	-	-	1.83E-03
TNF signalling pathway	04668	-	-	-	1.83E-03
Type I diabetes mellitus	04940	-	-	-	4.43E-03
African trypanosomiasis	05143	-	-	-	6.91E-03
Allograft rejection	05330	-	-	-	8.62E-03
Legionellosis	05134	-	-	-	9.35E-03
NF-κB signalling pathway	04064	-	-	-	1.07E-02
Tuberculosis	05152	-	-	-	4.56E-02

FDR <0.05. Fold change < –1.5 or >1.5.

**Figure 3:**
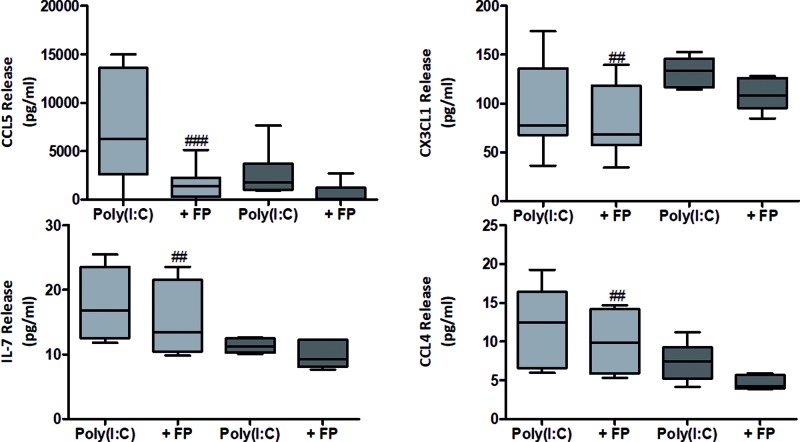
Effect of FP on poly(I:C)-induced inflammatory mediator signatures from ASMCs from healthy volunteers or chronic cough patients ASMCs from healthy volunteers (n=11, light grey) and chronic cough patients (n=6, dark grey) were treated with FP (10−8 M) for 2 h prior to 18 h stimulation with 5 μg/ml poly(I:C) or were left untreated (NT). Data are presented as mean ± S.D.; ##P<0.01, ###P<0.001 compared to poly(I:C).μg/ml poly(I:C) or were left untreated (NT). Data are presented as mean ± S.D.; ##P<0.01, ###P<0.001 compared to poly(I:C).

FP had a differential effect on poly(I:C)-induced mediator release from chronic cough and healthy non-cough volunteers (Figure 3, Supplementary Table S3). FP significantly inhibited poly(I:C)-induced CCL5, CX3CL1, IL-7 and CCL4 release from ASMCs from healthy non-cough volunteers (Figure 3, Supplementary Table S3). Conversely, only poly(I:C)-induced MCP-1 and TNFβ release was inhibited by FP in ASMCs from patients with chronic cough (Supplementary Table S3). Overall, there is a reduced ability of FP to suppress inflammatory gene and protein expression in ASMCs from patients with chronic cough compared with healthy controls.

## Discussion

We report that ASMCs from chronic cough patients have an enhanced infection response and a reduced innate immune and inflammatory response to poly(I:C) stimulation compared with cells from healthy non-cough volunteers. Pathway analysis also highlighted the differential effect of the inhaled glucocorticoid FP on inflammatory, innate immune/antiviral, oxidant stress and metabolic/mitochondrial function between cells from chronic cough patients and healthy volunteers. ASMCs from chronic cough patients also have a reduced response to FP compared with that seen in cells from healthy non-cough subjects.

This is the first report that highlights differences in gene expression and mediator release in ASMCs from chronic cough patients compared with healthy non-cough controls. Poly(I:C) stimulation up regulated a number of interferon-associated genes, such as *CXCL11* (interferon-inducible protein 9), *CXCL10* (interferon γ-induced protein 10) and *RSAD2* (radical S-adenosyl methionine domain containing 2) as well as *OAS2*, a gene that encodes a member of the 2-5A synthetase family, which has been involved in the innate immune response to viral infection [19,20]. This response is greater in cells from chronic cough patients than the healthy non-cough volunteers. In contrast, pathway analysis revealed that inflammatory pathways such as TNF signalling pathway, rheumatoid arthritis and NF-κB signalling pathway were reduced after poly(I:C) stimulation in chronic cough ASMCs compared with healthy non-cough subjects. This supports the concept of an impaired inflammatory response of chronic cough ASMCs to poly(I:C) stimulation as reflected in the inflammatory mediator release by these cells.

Impaired innate immune responses to infection have been reported previously in asthma. Asthmatic bronchial epithelial cells have a deficient IL-6 and CCL5 induction in response to rhinovirus-16 infection compared with cells from healthy volunteers [21]. Altered innate immune responses particularly TNFα release in response to the bacterial and viral ligands TLR4 and TLR7 have been reported in neonatal monocytes [22]. How this impairment in the innate immune function of the ASMCs link to chronic cough mechanisms is unclear but this may allow viruses to replicate more readily allowing viruses such as rhinoviruses to interact directly with epithelial nerve endings. Cross-talk featuring innate immune mediators between airway structural cells is becoming increasingly recognized as an important mechanism of disease [23].

On the other hand, the increased oxidative stress pathways at baseline and induced by poly(I:C) in ASMC from chronic cough patients is of interest. It has been previously reported that increased airway oxidative stress is associated with chronic cough [24]. Recent work has indicated that oxidative stress, in particular downstream of mitochondrial dysfunction, activates airway nociceptive sensory nerves that may contribute to an excessive cough reflex [25]. One of the mechanisms underlying oxidative stress is through the increased expression of transforming growth factor β (TGFβ) levels in BALF and in the bronchial mucosa and in particular ASMCs and the airway epithelium of patients with chronic cough [26]. TGFβ causes an oxidant–antioxidant imbalance in ASMCs and plays a major role in the redox-dependent pathways as it regulates antioxidant responses in ASMCs [17,27].

Although corticosteroids are not effective in controlling chronic cough of non-asthmatic origin, it was interesting to note that FP-treated ASMCs resulted in DEGs that have been previously linked to steroid responsiveness and inflammation, such as *FKBP5* [28,29], *DUSP1* [28,30], TSC22 domain family member 3 (*TSC22D3*) [28,29,31], period circadian clock (*PER1*) [28,32,33] and Kruppel-like factor 15 (*KLF15*) [28,34]. FP caused fewer changes in gene expression in ASMC from chronic cough patients than in healthy non-cough volunteers, which may be linked to the general lack of efficacy of corticosteroid treatment in chronic cough of non-asthmatic origin [1].

The differential expression of chemokine release that we have identified in the present study might affect infiltrating cell recruitment and have downstream effects on ASMC function. It is evident that the presence of infiltrating immune cells in close association with ASMCs modifies ASMC function and corticosteroid responses in asthma [16]. We have found that although there was an increase in mast cells in the airways submucosa of patients with chronic cough compared with subjects with no cough, there was no increase in the mast cells in the ASM compartment [7,11]. Therefore, it is unlikely that there is an increase in the direct interaction of immune/inflammatory cells with ASMCs in chronic cough. However, the increased number of mast cells in the submucosa may result in an increased potential for interactions with epithelial nerves which have increased transient receptor potential cation channel subfamily V member 1 (TRPV1) expression [35] that could underlie the increased sensitivity of the cough reflex found in chronic cough.

There are some limitations to the present study. The use of poly(I:C) as a stimulus may not replicate all the effects of live virus. In mitigation, we see activation of key viral pathways with poly(I:C) in these cells at the gene and protein level. The comparison of the effects of rhinovirus in disease models is often difficult due to the use of different isolates obtained from patients rather than the use of a GMP virus for example, and that the dose of virus often used to infect cells *in vitro* results in extensive cell death which is not observed *in vivo*. The mechanism underlying the altered response of ASMC cells from cough patients has not been elucidated. The cells were cultured over 4–6 passages which suggests that some degree of epigenetic reprogramming may exist. To our knowledge, differences in miRNA expression, DNA methylation status or histone marks have not been studied in patients with chronic cough. In addition, the use of a single 2 h pretreatment time point for FP as well as a single time point to analyse gene expression profiles may reduce the information about the effects observed. We have previously shown that 2 h pretreatment of ASMCs with dexamethasone significantly inhibited TNFα-induced cytokine release in ASMCs from healthy volunteers [15]. Further studies are needed to evaluate the time course of FP and stimulus effects on the transcriptome in ASMCs from chronic cough and healthy non-cough volunteers. Furthermore, a wider protein/mediator panel would aid the understanding of the impaired immune response seen in the chronic cough ASMCs.

**Figure 4:**
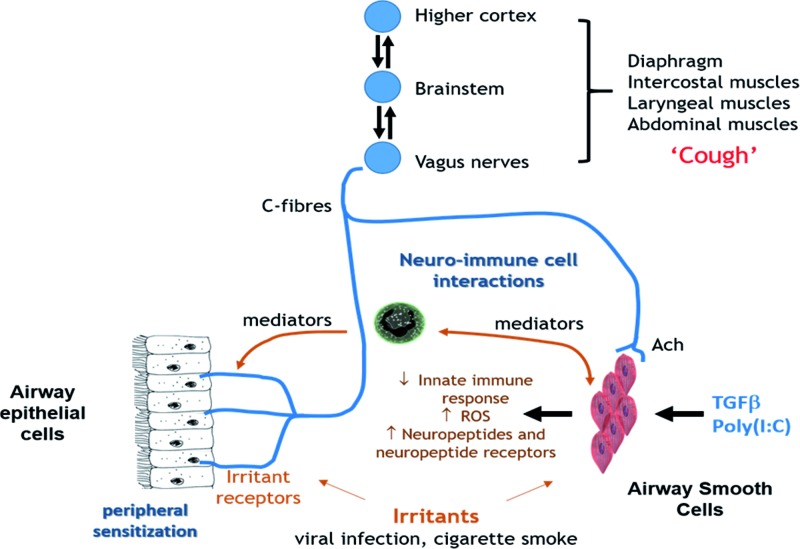
Schematic diagram of abnormal innate immune mechanisms in ASMCs which may affect chronic idiopathic cough Schematic presentation of interrelationships between major components in cough reflex pathway, particularly in relation to neuro–immune interaction. Inhaled triggers such as viral or bacterial infection or cigarette smoke stimulate both irritant receptors on c-fibres and immune cells within the airways. Cross-talk between activated sensory neurons and resident and infiltrating immune cells (neuro–immune cell interactions) occurs through the release of mediators. Enhanced activation of inflammatory responses and of oxidative stress (reactive oxygen species, ROS) and reduced innate immune mediator expression in ASMCs leads to the up-regulation of cough responses (peripheral sensitization) involving increased viral replication and activation of noci sensory nerves. Further interactions are mediated by mediators and receptors between the two systems. Stimulation of ASM cells by TGFβ, for example enhances the expression of neuropeptides and neuropeptide receptors in these cells and increases oxidative stress. The central cough generator then establishes and co-ordinates the output to the muscles that cause cough and to ASM cells. The responses in cough patients are less responsive to inhaled corticosteroids than ASMCs from healthy non-cough subjects.

We have shown for the first time that the baseline and stimulated innate immune response to infection in ASMCs is deficient in patients with chronic cough while oxidative stress pathways are enhanced. Poly(I:C) induces the expression of neuropeptides and receptors implicated in sensory nerve activation or cough equally in cells from chronic cough and healthy non-cough subjects. In addition, the reduced inflammatory response in cough ASMCs, the lack of sensitivity to FP and the enhanced expression of signatures relating to neuropeptides and neuronal ligands suggests that ASMCs may be important in local neuroinflammatory responses. An altered innate immune response particularly in response to poly(I:C) in ASMCs may have profound consequences on airway function and cough (Figure 4). The induction of the cough responses provoked by respiratory viruses, a common cause of cough, may be mediated by airway structural cells such as ASMCs as well as by infiltrating immune cells and nerves.

## Perspectives

- Chronic cough is associated with airway inflammation and remodelling which involves abnormal ASMC function. We aimed to determine whether the ASMC transcriptome is altered in chronic cough.
- Although there were no DEGs at baseline, ASMCs from chronic cough subjects had enhanced activation of viral response pathways in response to poly(I:C) compared with healthy subjects, reduced activation of pathways involved in chronic inflammation and an equivalent activation of neuroregulatory genes. ASMCs from cough patients also displayed a reduced responsiveness to FP.
- Enhanced viral responses, reduced innate immunity and an attenuated corticosteroid response of ASMC from patients with chronic cough may underlie the induction of cough triggered by respiratory viruses.

## Supporting information

**Table S1:** Top differentially expressed genes of cough ASMCs compared to healthy non-cough ASMCs at baseline.

**Table S2:** Top differentially expressed genes from ASMCs from chronic cough or healthy non-cough volunteers after poly(I:C) stimulation.

**Table S3:** Inflammatory mediator release from ASMCs after poly(I:C) stimulation in the presence or absence of fluticasone propionate (FP).

**Table S4:** Top differentially expressed genes from ASMCs from chronic cough or healthy non-cough volunteers after fluticasone propionate (FP) treatment.

**Table S5:** Top differentially expressed genes from poly(I:C)-stimulated ASMCs pretreated with fluticasone propionate (FP).

**Supplementary Table S6:** U-BIOPRED contributors

## Abbreviations

ASMC: airway smooth muscle cell
BALF: bronchoalveolar lavage fluid
CCL: chemokine (C–C motif) ligand
CXCL: chemokine (C–X–C motif) ligand
DEG: differentially expressed gene
DMEM: Dulbecco’s modified Eagle’s medium
DUSP1: dual specificity phosphatase 1
EGF: epidermal growth factor
FDR: false discovery rate
FKBP5: FK506 binding protein 5
FP: fluticasone propionate
IL: interleukin
JAK-STAT: Janus kinase/signal transducers and activators of transcription
MCP-1: monocyte chemotactic protein 1
NMDA: N-methyl-D-aspartate
PG: prostaglandin
PER1: period circadian clock
poly(I:C): polyinosinic:polycytidylic acid
QC: quality control
RBH: Royal Brompton and Harefield
TGFβ: transforming growth factor β
Th1: Type 1 T-helper cell
Th2: Type 2 T-helper cell
TLR: Toll-like receptor
TNFα: tumour necrosis factor α
TSC22D3: TSC22 domain family member 3
