# Supplementary material for: Impaired innate immune gene profiling in airway smooth muscle cells from chronic cough patients
Source: Biosci Rep. 2017 Nov 15;37(6):BSR20171090. doi: 10.1042/BSR20171090 (PMC5686396; doi:10.1042/BSR20171090)
Supplement: Supplementary file 1 [file bsr20171090_Supp1.pdf]

Supplementary material

Impaired innate immunity in airway smooth muscle cells from chronic cough patients

Christos Rossios[1,2], Stelios Pavlides[3], David Gibeon[1,2], Sharon Mumby[1,2], Andrew Durham[1,2], Oluwaseun Ojo[1,2], Daniel Horowitz[4], Matt Loza[4], Fred Baribaud[4], Navin Rao[4], Kian Fan Chung[1,2], Ian M Adcock[1,2,*] on behalf of U-BIOPRED WP6 study group[$]

Table S1. Top differentially expressed genes of cough ASMCs compared to healthy non-cough ASMCs at baseline.

Gene Symbol	Gene Title	Fold change[#]
ASPN	Asporin	3.27
PDE4DIP	Phosphodiesterase 4D interacting protein	3.13
IGJ	Immunoglobulin J polypeptide, linker protein for immunoglobulin alpha and mu polypeptides	2.80
LMCD1	LIM and cysteine-rich domains 1	2.43
LINC01140	Long intergenic non-protein coding RNA 1140	2.42
P2RY1	Purinergic receptor P2Y, G-protein coupled, 1	2.39
MAP2	Microtubule-associated protein 2	2.37
PLCXD3	Phosphatidylinositol-specific phospholipase C, X domain containing 3	2.31
MGP	Matrix Gla protein	2.23
ANKRD29	Ankyrin repeat domain 29	2.19
DHRS3	Dehydrogenase/reductase (SDR family) member 3	2.06
CLEC3B	C-type lectin domain family 3, member B	2.06
GULP1	GULP, engulfment adaptor PTB domain containing 1	2.02
GPRC5A	G protein-coupled receptor, class C, group 5, member A	1.99
HIST1H2AG	Histone cluster 1, H2ag	1.93
IQCA1	IQ motif containing with AAA domain 1	1.91
NEGR1	Neuronal growth regulator 1	1.91
MAF	v-Maf avian musculoaponeurotic fibrosarcoma oncogene homolog	1.89
SIPA1L2	Signal-induced proliferation-associated 1 like 2	1.86
PRG4	Proteoglycan 4	1.84
LINC01133	Long intergenic non-protein coding RNA 1133	1.81
LGI1	Leucine-rich, glioma inactivated 1	1.78
MEX3B	Mex-3 RNA binding family member B	1.77
UHRF1	Ubiquitin-like with PHD and ring finger domains 1	1.77

SLC35E2	Solute carrier family 35, member E2	1.73
NAAA	N-acylethanolamine acid amidase	1.73
FGF11	Fibroblast growth factor 11	1.72
C8orf48	Chromosome 8 open reading frame 48	1.72
EFCAB6	EF-hand calcium binding domain 6	1.70
PEX13	Peroxisomal biogenesis factor 13	1.70
GPR21	G protein-coupled receptor 21	-1.60
NKX1-1	NK1 homeobox 1	-1.60
UTP6	UTP6, small subunit (SSU) processome component, homolog (yeast)	-1.60
LIMS1	LIM and senescent cell antigen-like domains 1	-1.62
VNN1	Vanin 1	-1.62
FOXP1	Forkhead box P1	-1.62
KANSL1	KAT8 regulatory NSL complex subunit 1	-1.62
CAMK4	Calcium/calmodulin-dependent protein kinase IV	-1.63
ST3GAL3	ST3 beta-galactoside alpha-2,3-sialyltransferase 3	-1.64
DRAXIN	Dorsal inhibitory axon guidance protein	-1.65
GAFA3	FGF-2 activity-associated protein 3	-1.68
PDXDC1	Pyridoxal-dependent decarboxylase domain containing 1	-1.68
TRAF3IP2-AS1	TRAF3IP2 antisense RNA 1	-1.72
CYYR1	Cysteine/tyrosine-rich 1	-1.74
PPARGC1A	Peroxisome proliferator-activated receptor gamma, coactivator 1 alpha	-1.76
GAREM	GRB2 associated, regulator of MAPK1	-1.76
PLD1	Phospholipase D1, phosphatidylcholine-specific	-1.78
TTC14	Tetratricopeptide repeat domain 14	-1.79
ZNF678	Zinc finger protein 678	-1.80
PDXDC1	Pyridoxal-dependent decarboxylase domain-containing protein 1-like	-1.80
ZNRF2P1	Zinc and ring finger 2 pseudogene 1	-1.81
RGS4	Regulator of G-protein signaling 4	-1.82
COL4A1	Collagen, type IV, alpha 1	-1.82
KCNS2	Potassium voltage-gated channel, delayed-rectifier, subfamily S, member 2	-1.85
ST6GALNAC5	ST6 (alpha-N-acetyl-neuraminyl-2,3-beta-galactosyl-1,3)-N-acetylgalactosaminide alpha-2,6-sialyltransferase 5	-1.86
EDNRA	Endothelin receptor type A	-1.94
PLAT	Plasminogen activator, tissue	-1.97
NR4A2	Nuclear receptor subfamily 4, group A, member 2	-2.08
CXCL2	Chemokine (C-X-C motif) ligand 2	-3.27
CLDN1	Claudin 1	-3.29

#raw p value p<0.05, Fold change <-1.5 or >1.5.

Table S2. Top differentially expressed genes from ASMCs from chronic cough or healthy non-cough volunteers after poly(I:C) stimulation.

Gene Symbol	Gene Title	Cough*	Non-cough*
		Fold Change (FDR <.05)	
CXCL11	Chemokine (C-X-C motif) ligand 11	130.27	96.73
RSAD2	Radical S-adenosyl methionine domain containing 2	92.43	61.79
CCL5	Chemokine (C-C motif) ligand 5	78.19	56.96
CXCL10	Chemokine (C-X-C motif) ligand 10	68.68	49.55
OAS1	2'-5'-oligoadenylate synthetase 1, 40/46kDa	68.29	49.29
CMPK2	Cytidine monophosphate (UMP-CMP) kinase 2, mitochondrial	51.61	29.67
OAS2	2'-5'-oligoadenylate synthetase 2, 69/71kDa	39.51	18.90
MX1	Myxovirus (influenza virus) resistance 1, interferon-inducible protein p78 (mouse)	37.33	27.28
IFI44L	Interferon-induced protein 44-like	34.00	28.55
MX2	Myxovirus (influenza virus) resistance 2 (mouse)	31.55	25.92
OAS3	2'-5'-oligoadenylate synthetase 3, 100kDa	26.65	16.07
GBP4	Guanylate binding protein 4	21.91	19.90
OASL	2'-5'-oligoadenylate synthetase-like	21.23	19.82
IDO1	Indoleamine 2,3-dioxygenase 1	21.21	26.08
HERC5	HECT and RLD domain containing E3 ubiquitin protein ligase 5	16.95	9.92
IFIT2	Interferon-induced protein with tetratricopeptide repeats 2	16.45	16.37
IFIT1	Interferon-induced protein with tetratricopeptide repeats 1	15.16	15.28
IFI44	Interferon-induced protein 44	14.83	12.72
EPSTI1	Epithelial stromal interaction 1 (breast)	13.19	12.13
GBP1	Guanylate binding protein 1, interferon-inducible	12.64	12.70
HERC6	HECT and RLD domain containing E3 ubiquitin protein ligase family member 6	12.52	11.76
IFI6	Interferon, alpha-inducible protein 6	12.36	9.82
ISG15	ISG15 ubiquitin-like modifier	12.35	11.03
IFIT3	Interferon-induced protein with tetratricopeptide repeats 3	12.09	13.19
IFIH1	Interferon induced with helicase C domain 1	11.76	12.51
CTSS	Cathepsin S	11.61	8.40
LIPA	lipase A, lysosomal acid, cholesterol esterase	11.59	13.33
CXCL2	Chemokine (C-X-C motif) ligand 2	11.58	4.11
CCL8	Chemokine (C-C motif) ligand 8	11.03	22.25
USP18	Ubiquitin specific peptidase 18	10.51	8.95

RPS2	Ribosomal protein S2	-1.48	-1.51
MYL6B	Myosin, light chain 6B, alkali, smooth muscle and non-muscle	-1.51	-1.49
RPL4	Ribosomal protein L4	-1.51	-1.58
LTA4H	Leukotriene A4 hydrolase	-1.53	-1.48
RPL22	Ribosomal protein L22	-1.53	-1.64
EEF1G	Eukaryotic translation elongation factor 1 gamma	-1.59	-1.70
RPL5	ribosomal protein L5	-1.59	-1.61
ACBD4	Acyl-CoA binding domain containing 4	-1.66	-1.14$
RPS3	Ribosomal protein S3	-1.68	-1.60
SLC25A6	Solute carrier family 25 (mitochondrial carrier; adenine nucleotide translocator), member 6	-1.68	-1.84
HLCS	Holocarboxylase synthetase (biotin-(proprionyl-CoA-carboxylase (ATP-hydrolysing)) ligase)	-1.69	-1.36
RNU86	RNA, U86 small nucleolar	-1.73	-1.87
BAAT	Bile acid CoA:amino acid N-acyltransferase	-1.75	1.11$
EIF3F	Eukaryotic translation initiation factor 3, subunit F	-1.77	-1.97
LRRC75A-AS1	LRRC75A antisense RNA 1	-1.77	-1.93
EIF3L	Eukaryotic translation initiation factor 3, subunit L	-1.80	-1.92
RPL3	Ribosomal protein L3	-1.81	-1.84
RPLP0	Ribosomal protein, large, P0	-1.85	-2.00
EEF1B2	Eukaryotic translation elongation factor 1 beta 2	-1.86	-1.60
VPS37D	Vacuolar protein sorting 37 homolog D (S. cerevisiae)	-1.92	-1.48$
EIF4B	Eukaryotic translation initiation factor 4B	-1.92	-2.01
PARVA	Parvin, alpha	-1.96	-2.23
MTA3	Metastasis associated 1 family, member 3	-2.07	-1.46
AGBL5	ATP/GTP binding protein-like 5	-2.15	-2.39
AGO1	Argonaute RISC catalytic component 1	-2.18	-1.95
CERK	Ceramide kinase	-2.22	-2.00
BCAT2	Branched chain amino-acid transaminase 2, mitochondrial	-2.22	-2.20
NT5DC2	5'-nucleotidase domain containing 2	-2.40	-2.27
MEX3B	Mex-3 RNA binding family member B	-2.52	-1.49$
DANCR	Differentiation antagonizing non-protein coding RNA	-2.69	-2.36

* Significant FDR p<0.05, fold change-<1.5 or >1.5; $: Non-significant

Table S3. Inflammatory mediator release from ASMCs after poly(I:C) stimulation in the presence or absence of fluticasone propionate (FP).

Analyte (pg/ml)	Non-cough		Cough	
	Poly(I:C) (5≒g/ml)		Poly(I:C) (5≒g/ml)	
	-FP	+FP	-FP	+FP
**IFNγ**	6.8±2.4	5.0±2.4	6.7±2.9***	1.6±2.4
**IL-1α**	13.1±3.8***	11.5±3.9##	8.2±0.8***	7.1±0.7#
**IL-1RA**	47.5±15.2***	33.6±10.3###	41.0±5.3***	29.0±7.8#
**IL-6**	5568±2882**	997±959##	2333±2155***	411±436#
**TNFα**	nd	nd	nd	nd
**TNFβ**	11.7±1.1***	9.9±1.1	9.5±0.6***	7.9±0.6#
**IFNα2**	22.8±4.2***	19.1±3.9#	20.0±2.0***	16.3±1.4#
**IL-4**	19.2±4.7***	15.2±3.6##	19.1±2.0***	15.6±1.5#
**IL-13**	7.6±1.1***	6.3±0.9#	5.4±0.6***	4.2±0.3#
**IL-10**	4.9±1.4*	3.6±1.5#	4.7±0.6***	3.2±1.3#
**IL-12p40**	12.1±1.5***	10.1±1.3##	11.4±1.2***	8.4±1.3#
**IL-12p70**	3.7±2.5	2.4±2.2	4.8±0.7***	3.3±1.4
**CXCL10/IP-10**	12895±5015***	7979±6614###	7866±1568***	354±164
**CCL3/MIP-1α**	9.9±9.6	3.3±4.0##	18.8±20.4***	4.8±1.7#
**CCL4/MIP-1β**	11.9±5.3***	9.9±3.9##	7.4±2.5***	4.6±0.9
**CCL2/MCP-1**	9748±4422	8555±4251	8102±930***	4929±2656#
**CCL7/MCP-3**	2381±1847***	173±240###	1301±992***	41.1±21.2##
**CXCL1/GRO**	5981±2741***	4609±3008	3265±3123***	1398±1474
**CCL5/RANTES**	7052±5513**	1672±1614###	2596±2550***	613±1060
**CCL11/Eotaxin**	3831±3431*	2685±3063	4286±4701***	1848±3078
**CX3CL1/Fractalkine**	101±45**	85.0±36.7##	132.3±14.9***	108.8±16.8
**CXCL8**	6947±3297*	2555±2126	3642±2070***	1174±820
**G-CSF**	837±833**	198±208	386±536***	102.2±147.8
**GM-CSF**	45.3±68.5	3.5±5.3#	19.4±20.5***	0.64±0
**EGF**	5.9±1.0**	5.4±1.0	5.5±1.9***	4.3±0.3#
**VEGF**	142.3±78.9	87.0±15.3##	107.1±17.1***	76.0±4.6

**IL-7**	18.0±5.7*	15.5±5.5	11.3±1.1***	9.8±2.0

* $p<0.05$ vs. baseline; **$p<0.01$ vs. baseline; ***$p<0.001$ vs. baseline; $^{\#}$ $p<0.05$ vs. stimulation; $^{\#\#}$ $p<0.01$ vs. stimulation; $^{\#\#\#}$ $p<0.001$ vs. stimulation

Table S4. Top differentially expressed genes from ASMCs from chronic cough or healthy non-cough volunteers after fluticasone propionate (FP) treatment.

Gene Symbol	Gene Title	Cough*	Non-cough*
		Fold Change (FDR <0.05)	
FAM107A	Family with sequence similarity 107, member A	22.53	12.50
FKBP5	FK506 binding protein 5	11.33	13.88
SPARCL1	SPARC-like 1 (hevin)	6.99	3.86
MAOA	Monoamine oxidase A	5.56	6.47
RASL11B	RAS-like, family 11, member B	5.49	7.39
ZBTB16	Zinc finger and BTB domain containing 16	5.47	5.49
HSD11B1	Hydroxysteroid (11-beta) dehydrogenase 1	4.97	3.09
TSC22D3	TSC22 domain family, member 3	4.65	5.29
GLUL	Glutamate-ammonia ligase	3.97	4.04
FOXO1	Forkhead box O1	3.40	2.98
DUSP1	Dual specificity phosphatase 1	3.30	2.88
SAMHD1	SAM domain and HD domain 1	3.29	3.64
MIR6883	microRNA 6883	3.17	2.50
MT1X	Metallothionein 1X	2.55	2.00
KLF9	Kruppel-like factor 9	2.34	2.51
MT1E	Metallothionein 1E	2.28	1.73
JADE1	Jade family PHD finger 1	2.22	2.31
MT1F	Metallothionein 1F	2.17	1.73
MT1H	Metallothionein 1H	2.17	1.62
UGT3A1	UDP glycosyltransferase 3 family, polypeptide A1	2.01	1.10$
C1orf177	Chromosome 1 open reading frame 177	1.98	-1.06$
MT2A	Metallothionein 2A	1.91	1.49
HBZ	Hemoglobin, zeta	1.89	-1.05$
PHC2	Polyhomeotic homolog 2 (Drosophila)	1.89	1.74
PGLYRP1	Peptidoglycan recognition protein 1	1.81	1.13$
BSPRY	B-box and SPRY domain containing	1.71	1.19$
PQLC2	PQ loop repeat containing 2	1.60	-1.01$
FADS3	Fatty acid desaturase 3	1.60	1.44$
NR3C1	Nuclear receptor subfamily 3, group C, member 1 (glucocorticoid receptor)	-1.84	-1.40$
NF1	Neurofibromin 1	-2.04	-1.16$
NR1D2	Nuclear receptor subfamily 1, group D, member 2	-2.18	-2.38
ZNF827	Zinc finger protein 827	-2.18	-1.46$
MEX3B	Mex-3 RNA binding family member B	-2.36	-1.31$
PER2	Period circadian clock 2	-2.46	-2.26

TEF	Thyrotrophic embryonic factor	-2.83	-2.17
DACH1	Dachshund family transcription factor 1	-3.42	-2.11$
SLC8A1	Solute carrier family 8 (sodium/calcium exchanger), member 1	-4.20	-3.63
PER3	Period circadian clock 3	-5.90	-3.88

Fold change <-1.5 or >1.5. $: non-significant

Table S5: Top differentially expressed genes from poly(I:C)-stimulated ASMCs pre-treated with fluticasone propionate (FP).

Gene Symbol	Gene Title	Cough*	Non-cough*
		Fold Change (FDR <0.05)	
ZBTB16	Zinc finger and BTB domain containing 16	5.80	5.74
FAM107A	Family with sequence similarity 107, member A	5.39	6.12
DANCR	Differentiation antagonizing non-protein coding RNA	3.79	4.20
MAOA	Monoamine oxidase A	3.68	8.14
FKBP5	FK506 binding protein 5	3.56	5.92
FOXO1	Forkhead box O1	3.11	3.40
GLUL	Glutamate-ammonia ligase	2.63	2.84
KLF15	Kruppel-like factor 15	2.47	1.59$
MRPS23	Mitochondrial ribosomal protein S23	2.43	2.06
NRG1-IT1	NRG1 intronic transcript 1 (non-protein coding)	2.19	-1.07$
FAM46B	Family with sequence similarity 46, member B	2.11	1.67
CYP19A1	Cytochrome P450, family 19, subfamily A, polypeptide 1	2.02	1.92
GLULP4	Glutamate-ammonia ligase (glutamine synthetase) pseudogene 4	2.01	1.35$
WNT10A	Wingless-type MMTV integration site family, member 10A	1.97	1.12$
OR7E14P	Olfactory receptor, family 7, subfamily E, member 14 pseudogene	1.97	1.03$
CCDC136	Coiled-coil domain containing 136	1.95	-1.00$
PLB1	Phospholipase B1	1.91	1.10$
NXF2	Nuclear RNA export factor 2	1.87	-1.19$
CHFR	Checkpoint with forkhead and ring finger domains, E3 ubiquitin protein ligase	1.81	1.03$
TGM3	Transglutaminase 3	1.80	-1.02$
PRX	Periaxin	1.78	1.14$
LINC00659	Long intergenic non-protein coding RNA 659	1.76	-1.14$
SLFNL1	Schlafen-like 1	1.68	1.20$
TTPA	Tocopherol (alpha) transfer protein	1.66	1.01$
PABPC4	Poly(A) binding protein, cytoplasmic 4 (inducible form)	1.64	1.35
IGLJ3	Immunoglobulin lambda joining 3	1.62	-1.05$
TCEAL4	Transcription elongation factor A (SII)-like 4	1.62	2.04
PHC2	Polyhomeotic homolog 2 (Drosophila)	1.55	1.74
PPM1K	Protein phosphatase, Mg2+/Mn2+ dependent, 1K	-3.35	-1.78$
PER3	Period circadian clock 3	-4.82	-6.12
IFIT3	Interferon-induced protein with tetratricopeptide repeats 3	-4.82	-3.78
PLA2G4A	Phospholipase A2, group IVA (cytosolic, calcium-dependent)	-5.15	-8.96

TRIM5	Tripartite motif containing 5	-5.25	-4.49
ACTN2	Actinin, alpha 2	-5.32	-4.96
RET	Ret proto-oncogene	-5.32	-4.75
PARP14	Poly (ADP-ribose) polymerase family, member 14	-5.37	-3.20
IL18BP	Interleukin 18 binding protein	-5.56	-4.66
TRIM14	Tripartite motif containing 14	-6.02	-2.81
LIPA	Lipase A, lysosomal acid, cholesterol esterase	-6.44	-5.34
USP18	Ubiquitin specific peptidase 18	-6.45	-3.26
HERC5	HECT and RLD domain containing E3 ubiquitin protein ligase 5	-6.50	-2.85
OASL	2'-5'-oligoadenylate synthetase-like	-6.60	-4.94
HERC6	HECT and RLD domain containing E3 ubiquitin protein ligase family member 6	-6.77	-2.58
IFIH1	Interferon induced with helicase C domain 1	-6.90	-4.69
CD274	CD274 molecule	-7.51	-9.04
OAS1	2'-5'-oligoadenylate synthetase 1, 40/46kDa	-7.54	-3.45
IFIT2	Interferon-induced protein with tetratricopeptide repeats 2	-8.17	-4.99
IFI44	Interferon-induced protein 44	-8.33	-2.65[$]
TLR3	Toll-like receptor 3	-8.37	-3.67
IDO1	Indoleamine 2,3-dioxygenase 1	-8.56	-7.81
PTGS2	Prostaglandin-endoperoxide synthase 2 (prostaglandin G/H synthase and cyclooxygenase)	-8.92	-21.84
GBP4	Guanylate binding protein 4	-9.10	-7.83
ISG20	Interferon stimulated exonuclease gene 20kDa	-9.16	-7.48
TNFSF13B	Tumour necrosis factor (ligand) superfamily, member 13b	-10.25	-5.77
CXCL10	Chemokine (C-X-C motif) ligand 10	-11.93	-10.39
TNFSF10	Tumour necrosis factor (ligand) superfamily, member 10	-12.98	-9.69
CCL8	Chemokine (C-C motif) ligand 8	-13.64	-15.33
RSAD2	Radical S-adenosyl methionine domain containing 2	-14.43	-5.13

* Significant FDR p<0.05, Fold change<-1.5 or >1.5; [$]: Non-significant.

Supplementary Table S6: U-BIOPRED contributors

The U-BIOPRED consortium wishes to acknowledge the help and expertise of the following individuals and groups without whom, the study would not have been possible:

**U-BIOPRED Supplementary authors**	
**Name**	**Affiliation**
Adcock I M	National Heart and Lung Institute, Imperial College, London, UK;
Ahmed H	European Institute for Systems Biology and Medicine, CNRS-ENS-UCBL-INSERM, Lyon, France;
Auffray C	European Institute for Systems Biology and Medicine, CNRS-ENS-UCBL-INSERM, Lyon, France;
Bakke P	Department of Clinical Science, University of Bergen, Bergen, Norway;
Bansal A T	Acclarogen Ltd, St. John's Innovation Centre, Cambridge, UK;
Baribaud F	Janssen R&D, USA;
Bates S	Respiratory Therapeutic Unit, GSK, London, UK;
Bel E H	Academic Medical Centre, University of Amsterdam, Amsterdam, The Netherlands;
Bigler J	*Previously Amgen Inc*
Bisgaard H	COPSAC, Copenhagen Prospective Studies on Asthma in Childhood, Herlev and Gentofte Hospital,<br>University of Copenhagen, Copenhagen, Denmark
Boedigheimer M J	Amgen Inc.; Thousand Oaks, USA
Bønnelykke K	COPSAC, Copenhagen Prospective Studies on Asthma in Childhood, Herlev and Gentofte<br>Hospital, University of Copenhagen, Copenhagen, Denmark;
Brandsma J	University of Southampton, Southampton, UK
Brinkman P	Academic Medical Centre, University of Amsterdam, Amsterdam, The Netherlands;
Bucchioni E	Chiesi Pharmaceuticals SPA, Parma, Italy
Burg D	Centre for Proteomic Research, Institute for Life Sciences, University of Southampton, Southampton, UK
Bush A	National Heart and Lung Institute, Imperial College, London, UK; Royal Brompton and Harefield NHS trust, UK
Caruso M	Dept. Clinical and Experimental Medicine, University of Catania,

	Catania, Italy;
Chaiboonchoe A	European Institute for Systems Biology and Medicine, CNRS-ENS-UCBL-INSERM, Lyon, France;
Chanez P	Assistance publique des Hôpitaux de Marseille - Clinique des bronches, allergies et sommeil, Aix Marseille Université, Marseille, France
Chung F K	National Heart and Lung Institute, Imperial College, London, UK;
Compton C H	Respiratory Therapeutic Unit, GSK, London, UK
Corfield J	Areteva R&D, Nottingham, UK;
D'Amico A	University of Rome 'Tor Vergata', Rome Italy;
Dahlen S E	Centre for Allergy Research, Karolinska Institutet, Stockholm, Sweden
De Meulder B	European Institute for Systems Biology and Medicine, CNRS-ENS-UCBL-INSERM, Lyon, France;
Djukanovic R	NIHR Southampton Respiratory Biomedical Research Unit and Clinical and Experimental Sciences, Southampton, UK;
Erpenbeck V J	Translational Medicine, Respiratory Profiling, Novartis Institutes for Biomedical Research, Basel, Switzerland;
Erzen D	Boehringer Ingelheim Pharma GmbH & Co. KG; Biberach, Germany
Fichtner K	Boehringer Ingelheim Pharma GmbH & Co. KG; Biberach, Germany
Fitch N	BioSci Consulting, Maasmechelen, Belgium;
Fleming L J	National Heart and Lung Institute, Imperial College, London, UK; Royal Brompton and Harefield NHS trust, UK
Formaggio E	*Previously CROMSOURCE, Verona Italy*
Fowler S J	Centre for Respiratory Medicine and Allergy, Institute of Inflammation and Repair, University of Manchester and University Hospital of South Manchester, Manchester Academic Health Sciences Centre, Manchester, United Kingdom
Frey U	University Children's Hospital, Basel, Switzerland;
Gahlemann M	Boehringer Ingelheim (Schweiz) GmbH,Basel, Switzerland;
Geiser T	Department of Respiratory Medicine, University Hospital Bern, Switzerland;
Guo Y	Data Science Institute, Imperial College, London, UK;
Hashimoto S	Academic Medical Centre, University of Amsterdam, Amsterdam, The Netherlands;
Haughney J	International Primary Care Respiratory Group, Aberdeen, Scotland;
Hedlin G	Dept. Women's and Children's Health & Centre for Allergy Research, Karolinska Institutet, Stockholm, Sweden;

Hekking P W	Academic Medical Centre, University of Amsterdam, Amsterdam, The Netherlands;
Higenbottam T	Allergy Therapeutics, West Sussex, UK;
Hohlfeld J M	Fraunhofer Institute for Toxicology and Experimental Medicine, Hannover, Germany
Holweg C	Respiratory and Allergy Diseases, Genentech, San Francisco, USA
Horváth I	Semmelweis University, Budapest, Hungary
Howarth P	NIHR Southampton Respiratory Biomedical Research Unit, Clinical and Experimental Sciences and Human Development and Health, Southampton, UK
James A J	Centre for Allergy Research, Karolinska Institutet, Stockholm, Sweden;
Knowles R	Arachos Pharma, Stevenge, UK;
Knox A J	Respiratory Research Unit, University of Nottingham, Nottingham, UK;
Krug N	Fraunhofer Institute for Toxicology and Experimental Medicine, Hannover, Germany;
Lefaudeux D	European Institute for Systems Biology and Medicine, CNRS-ENS-UCBL-INSERM, Lyon, France;
Loza M J	Janssen R&D, USA;
Lutter R	Academic Medical Centre, University of Amsterdam, Amsterdam, The Netherlands;
Manta A	Roche Diagnostics GmbH, Mannheim, Germany
Masefield S	European Lung Foundation, Sheffield, UK;
Matthews J G	Respiratory and Allergy Diseases, Genentech, San Francisco, USA;
Mazein A	European Institute for Systems Biology and Medicine, CNRS-ENS-UCBL-INSERM, Lyon, France
Meiser A	Data Science Institute, Imperial College, London, UK
Middelveld R J M	Centre for Allergy Research, Karolinska Institutet, Stockholm, Sweden
Miralpeix M	Almirall, Barcelona, Spain;
Montuschi P	Università Cattolica del Sacro Cuore, Milan, Italy;
Mores N	Università Cattolica del Sacro Cuore, Milan, Italy;
Murray C S	Centre for Respiratory Medicine and Allergy, Institute of Inflammation and Repair, University of Manchester and University Hospital of South Manchester, Manchester Academic Health Sciences Centre, Manchester, United Kingdom
Musial J	Dept. of Medicine, Jagiellonian University Medical College, Krakow, Poland

Myles D	Respiratory Therapeutic Unit, GSK, London, UK;
Pahus L	Assistance publique des Hôpitaux de Marseille, Clinique des bronches, allergies et sommeil<br>Espace Éthique Méditerranéen, Aix-Marseille Université, Marseille, France;
Pandis I	Data Science Institute, Imperial College, London, UK
Pavlidis S	National Heart and Lung Institute, Imperial College, London, UK
Powel P	European Lung Foundation, Sheffield, UK;
Praticò G	CROMSOURCE, Verona, Italy
Puig Valls M	CROMSOURCE, Barcelona, Spain
Rao N	Janssen R&D, USA;
Riley J	Respiratory Therapeutic Unit, GSK, London, UK;
Roberts A	Asthma UK, London, UK;
Roberts G	NIHR Southampton Respiratory Biomedical Research Unit, Clinical and Experimental Sciences and Human Development and Health, Southampton, UK;
Rowe A	Janssen R&D, UK;
Sandström T	Dept of Public Health and Clinical Medicine, Umeå University, Umeå, Sweden;
Seibold W	Boehringer Ingelheim Pharma GmbH, Biberach, Germany
Selby A	NIHR Southampton Respiratory Biomedical Research Unit, Clinical and Experimental Sciences and Human Development and Health, Southampton, UK;
Shaw D E	Respiratory Research Unit, University of Nottingham, UK;
Sigmund R	Boehringer Ingelheim Pharma GmbH & Co. KG; Biberach, Germany
Singer F	University Children's Hospital, Zurich, Switzerland;
Skipp P J	Centre for Proteomic Research, Institute for Life Sciences, University of Southampton, Southampton, UK
Sousa A R	Respiratory Therapeutic Unit, GSK, London, UK;
Sterk P J	Academic Medical Centre, University of Amsterdam, Amsterdam, The Netherlands;
Sun K	Data Science Institute, Imperial College, London, UK
Thornton B	MSD, USA
van Aalderen W M	Academic Medical Centre, University of Amsterdam, Amsterdam, The Netherlands;
van Geest M	AstraZeneca, Mölndal, Sweden;
Vestbo J	Centre for Respiratory Medicine and Allergy, Institute of Inflammation and Repair, University of Manchester and University

	Hospital of South Manchester, Manchester Academic Health Sciences Centre, Manchester, United Kingdom
Vissing N H	COPSAC, Copenhagen Prospective Studies on Asthma in Childhood, Herlev and Gentofte Hospital,<br><br>University of Copenhagen, Copenhagen, Denmark;
Wagener A H	Academic Medical Center Amsterdam, Amsterdam, The Netherlands
Wagers S S	BioSci Consulting, Maasmechelen, Belgium
Weiszhart Z	Semmelweis University, Budapest, Hungary;
Wheelock C E	Centre for Allergy Research, Karolinska Institutet, Stockholm, Sweden;
Wilson S J	Histochemistry Research Unit, Faculty of Medicine, University of Southampton, Southampton, UK;

Contributors

Aliprantis Antonios, Merck Research Laboratories, Boston, USA;
Allen David, North West Severe Asthma Network, Pennine Acute Hospital NHS Trust, UK
Alving Kjell, Dept Women's & Children's Health, Uppsala University, Uppsala, Sweden
Badorrek P, Fraunhofer ITEM; Hannover, Germany
Balgoma David, Centre for Allergy Research, Karolinska Institutet, Stockholm, Sweden
Ballereau S, European institute for Systems Biology and Medicine, University of Lyon, France
Barber Clair, NIHR Southampton Respiratory Biomedical Research Unit and Clinical and Experimental Sciences, Southampton, UK;
Batuwitage Manohara Kanangana, Data Science Institute, Imperial College, London, UK
Bautmans An, MSD, Brussels, Belgium
Bedding A, Roche Diagnostics GmbH, Mannheim, Germany
Behndig AF, Umeå University, Umea, Sweden
Beleta Jorge, Almirall S.A., Barcelona, Spain;
Berglind A, MSD, Brussels, Belgium
Berton A, AstraZeneca, Mölndal, Sweden
Bochenek Grazyna, II Department of Internal Medicine, Jagiellonian University Medical

College, Krakow, Poland;
Braun Armin, Fraunhofer Institute for Toxicology and Experimental Medicine, Hannover, Germany;
Campagna D, Department of Clinical and Experimental Medicine, University of Catania, Catania, Italy;
Carayannopoulos Leon, Previously at: MSD, USA;
Casaulta C, University Children’s Hospital of Bern, Switzerland
Chaleckis Romanas, Centre of Allergy Research, Karolinska Institutet, Stockholm, Sweden
Dahlén B, Karolinska University Hospital & Centre for Allergy Research, Karolinska Institutet, Stockholm, Sweden
Davison Timothy Janssen R&D, USA;
De Alba Jorge, Almirall S.A., Barcelona, Spain;
De Lepeleire Inge, MSD, Brussels, BE
Dekker Tamara, Academic Medical Centre, University of Amsterdam, Amsterdam, The Netherlands;
Delin Ingrid, Centre for Allergy Research, Karolinska Institutet, Stockholm, Sweden
Dennison P, NIHR Southampton Respiratory Biomedical Research Unit, Clinical and Experimental Sciences, NIHR-Wellcome Trust Clinical Research Facility, Faculty of Medicine, University of Southampton, Southampton, UK;
Dijkhuis Annemiek, Academic Medical Centre, University of Amsterdam, Amsterdam, The Netherlands;
Dodson Paul, AstraZeneca, Mölndal, Sweden
Draper Aleksandra, BioSci Consulting, Maasmechelen, Belgium;
Dyson K, CROMSOURCE; Stirling, UK
Edwards Jessica, Asthma UK, London, UK;
El Hadjam L, European Institute for Systems Biology and Medicine, University of Lyon
Emma Rosalia, Department of Clinical and Experimental Medicine, University of Catania, Catania, Italy;
Ericsson Magnus, Karolinska University Hospital, Stockholm, Sweden
Faulenbach C, Fraunhofer ITEM; Hannover, Germany
Flood Breda, European Federation of Allergy and Airways Diseases Patient’s Associations,

Brussels, Belgium
Galffy G, Semmelweis University, Budapest, Hungary;
Gallart Hector, Centre for Allergy Research, Karolinska Institutet, Stockholm, Sweden
Garissi D, Global Head Clinical Research Division, CROMSOURCE, Italy
Gent J, Royal Brompton and Harefield NHS Foundation Trust, London, UK;
Gerhardsson de Verdier M, AstraZeneca; Mölndal, Sweden;
Gibeon D, National Heart and Lung Institute, Imperial College, London, UK;
Gomez Cristina, Centre for Allergy Research, Karolinska Institutet, Stockholm, Sweden
Gove Kerry, NIHR Southampton Respiratory Biomedical Research Unit and Clinical and Experimental Sciences, Southampton, UK;
Gozzard Neil, UCB, Slough, UK;
Guillmant-Farry E, Royal Brompton Hospital, London, UK
Henriksson E, Karolinska University Hospital & Karolinska Institutet, Stockholm, Sweden
Hewitt Lorraine, NIHR Southampton Respiratory Biomedical Research Unit, Southampton, UK
Hoda U, Imperial College, London, UK
Hu Richard, Amgen Inc. Thousand Oaks, USA
Hu Sile, National Heart and Lung Institute, Imperial College, London, UK;
Hu X, Amgen Inc.; Thousand Oaks, USA
Jeyasingham E, UK Clinical Operations, GSK, Stockley Park, UK
Johnson K, Centre for respiratory medicine and allergy, Institute of Inflammation and repair, University Hospital of South Manchester, NHS Foundation Trust, Manchester, UK
Jullian N, European Institute for Systems Biology and Medicine, University of Lyon
Kamphuis Juliette, Longfonds, Amersfoort, The Netherlands;
Kennington Erika J., Asthma UK, London, UK;
Kerry Dyson, CromSource, Stirling, UK;
Kerry G, Centre for respiratory medicine and allergy, Institute of Inflammation and repair, University Hospital of South Manchester, NHS Foundation Trust, Manchester, UK

Klüglich M, Boehringer Ingelheim Pharma GmbH & Co. KG; Biberach, Germany
Knobel Hugo, Philips Research Laboratories, Eindhoven, The Netherlands;
Kolmert Johan, Centre for Allergy Research, Karolinska Institutet, Stockholm, Sweden
Konradsen J R, Dept. Women's and Children's Health & Centre for Allergy Research, Karolinska Institutet, Stockholm, Sweden
Kots Maxim, Chiesi Pharmaceuticals, SPA, Parma, Italy;
Kretsos Kosmas, UCB, Slough, UK
Krueger L, University Children's Hospital Bern, Switzerland
Kuo Scott, National Heart and Lung Institute, Imperial College, London, UK;
Kupczyk Maciej, Centre for Allergy Research, Karolinska Institutet, Stockholm, Sweden
Lambrecht Bart, University of Gent, Gent, Belgium;
Lantz A-S, Karolinska University Hospital & Centre for Allergy Research, Karolinska Institutet, Stockholm, Sweden
Larminie Christopher, GSK, London, UK
Larsson L X, AstraZeneca, Mölndal, Sweden
Latzin P, University Children's Hospital of Bern, Bern, Switzerland
Lazarinis N, Karolinska University Hospital & Karolinska Institutet, Stockholm, Sweden
Lemonnier N, European Institute for Systems Biology and Medicine, CNRS-ENS-UCBL-INSERM, Lyon, France
Lone-Latif Saeeda, Academic Medical Centre, University of Amsterdam, Amsterdam, The Netherlands;
Lowe L A, Centre for respiratory medicine and allergy, Institute of Inflammation and repair, University Hospital of South Manchester, NHS Foundation Trust, Manchester, UK
Manta Alexander, Roche Diagnostics GmbH, Mannheim, Germany
Marouzet Lisa, NIHR Southampton Respiratory Biomedical Research Unit, Southampton, UK
Martin Jane, NIHR Southampton Respiratory Biomedical Research Unit, Southampton, UK
Mathon Caroline, Centre of Allergy Research, Karolinska Institutet, Stockholm, Sweden
McEvoy L, University Hospital, Department of Pulmonary Medicine, Bern, Switzerland
Meah Sally, National Heart and Lung Institute, Imperial College, London, UK;

Menzies-Gow A, Royal Brompton and Harefield NHS Foundation Trust, London, UK;
Metcalf Leanne, Previously at: Asthma UK, London, UK;
Mikus Maria, Science for Life Laboratory & The Royal Institute of Technology, Stockholm, Sweden;
Monk Philip, Synairgen Research Ltd, Southampton, UK;
Naz Shama, Centre for Allergy Research, Karolinska Institutet, Stockholm, Sweden
Nething K, Boehringer Ingelheim Pharma GmbH & Co. KG; Biberach, Germany
Nicholas Ben, University of Southampton, Southampton, UK
Nihlén U, *Previously AstraZeneca; Mölndal, Sweden;*
Nilsson Peter, Science for Life Laboratory & The Royal Institute of Technology, Stockholm, Sweden;
Niven R, North West Severe Asthma Network, University Hospital South Manchester, UK
Nordlund B, Dept. Women's and Children's Health & Centre for Allergy Research, Karolinska Institutet, Stockholm, Sweden
Nsubuga S, Royal Brompton Hospital, London, UK
Östling Jörgen, AstraZeneca, Mölndal, Sweden;
Pacino Antonio, Lega Italiano Anti Fumo, Catania, Italy;
Palkonen Susanna, European Federation of Allergy and Airways Diseases Patient's Associations, Brussels, Belgium.
Pellet J, European Institute for Systems Biology and Medicine, CNRS-ENS-UCBL-INSERM, Lyon, France
Pennazza Giorgio, University of Rome 'Tor Vergata', Rome Italy;
Petrén Anne, Centre for Allergy Research, Karolinska Institutet, Stockholm, Sweden
Pink Sandy, NIHR Southampton Respiratory Biomedical Research Unit, Southampton, UK
Pison C, European Institute for Systems Biology and Medicine, CNRS-ENS-UCBL-INSERM, Lyon, France
Postle Anthony, University of Southampton, UK
Rahman-Amin Malayka, Previously at: Asthma UK, London, UK;
Ravanetti Lara, Academic Medical Centre, University of Amsterdam, Amsterdam, The Netherlands;

Ray Emma, NIHR Southampton Respiratory Biomedical Research Unit, Southampton, UK
Reinke Stacey, Centre for Allergy Research, Karolinska Institutet, Stockholm, Sweden
Reynolds Leanne, Previously at: Asthma UK, London, UK;
Riemann K, Boehringer Ingelheim Pharma GmbH & Co. KG; Biberach, Germany
Robberechts Martine, MSD, Brussels, Belgium
Rocha J P, Royal Brompton and Harefield NHS Foundation Trust
Rossios C, National Heart and Lung Institute, Imperial College, London, UK;
Russell Kirsty, National Heart and Lung Institute, Imperial College, London, UK;
Rutgers Michael, Longfonds, Amersfoort, The Netherlands;
Santini G, Università Cattolica del Sacro Cuore, Milan, Italy;
Santoninco Marco, University of Rome ‘Tor Vergata’, Rome Italy;
Saqi M, European Institute for Systems Biology and Medicine, CNRS-ENS-UCBL-INSERM, Lyon, France
Schoelch Corinna, Boehringer Ingelheim Pharma GmbH & Co. KG, Biberach, Germany
Schofield James P. R., Centre for Proteomic Research, Institute for Life Sciences, University of Southampton, Southampton, UK
Scott S, North West Severe Asthma Network, Countess of Chester Hospital, UK
Sehgal N, North West Severe Asthma Network; Pennine Acute Hospital NHS Trust
Sjödin Marcus, Centre for Allergy Research, Karolinska Institutet, Stockholm, Sweden
Smids Barbara, Academic Medical Centre, University of Amsterdam, Amsterdam, The Netherlands;
Smith Caroline, NIHR Southampton Respiratory Biomedical Research Unit, Southampton, UK
Smith Jessica, Asthma UK, London, UK;
Smith Katherine M., University of Nottingham, UK;
Söderman P, Dept. Women’s and Children’s Health, Karolinska Institutet, Stockholm, Sweden
Sogbesan A, Royal Brompton and Harefield NHS Foundation Trust, London, UK;
Spycher F, University Hospital Department of Pulmonary Medicine, Bern, Switzerland
Staykova Doroteya, University of Southampton, Southampton, UK

Stephan S, Centre for respiratory medicine and allergy, Institute of Inflammation and repair, University Hospital of South Manchester, NHS Foundation Trust, Manchester, UK
Stokholm J, University of Copenhagen and Danish Pediatric Asthma Centre Denmark
Strandberg K, Karolinska University Hospital & Karolinska Institutet, Stockholm, Sweden
Sunther M, Centre for respiratory medicine and allergy, Institute of Inflammation and repair, University Hospital of South Manchester, NHS Foundation Trust, Manchester, UK
Szentkereszty M, Semmelweis University, Budapest, Hungary;
Tamasi L, Semmelweis University, Budapest, Hungary;
Tariq K, NIHR Southampton Respiratory Biomedical Research Unit, Clinical and Experimental Sciences, NIHR-Wellcome Trust Clinical Research Facility, Faculty of Medicine, University of Southampton, Southampton, UK;
Thörngren John-Olof, Karolinska University Hospital, Stockholm, Sweden
Thorsen Jonathan, COPSAC, Copenhagen Prospective Studies on Asthma in Childhood, Herlev and Gentofte<br><br>Hospital, University of Copenhagen, Copenhagen, Denmark;
Valente S, Università Cattolica del Sacro Cuore, Milan, Italy;
van de Pol Marianne, Academic Medical Centre, University of Amsterdam, Amsterdam ,The Netherlands;
van Drunen C M, Academic Medical Centre, University of Amsterdam, Amsterdam, The Netherlands;
Van Eyll Jonathan, UCB, Slough, UK
Versnel Jenny, Previously at: Asthma UK, London, UK;
Vink Anton, Philips Research Laboratories, Eindhoven, The Netherlands;
von Garnier C, University Hospital Bern, Switzerland;
Vyas A, North west Severe Asthma Network, Lancashire Teaching Hospitals NHS Trust, UK
Wald Frans, Boehringer Ingelheim Pharma GmbH & Co. KG, Biberach, Germany
Walker Samantha, Asthma UK, London, UK;
Ward Jonathan, Histochemistry Research Unit, Faculty of Medicine, University of Southampton, Southampton, UK;
Wetzel Kristiane, Boehringer Ingelheim Pharma GmbH, Biberach, Germany

Wiegman Coen, National Heart and Lung Institute, Imperial College, London, UK;
Williams Siân, International Primary Care Respiratory Group, Aberdeen, Scotland;
Yang Xian, Data Science Institute, Imperial College, London, UK
Yeyasingham Elizabeth, UK Clinical Operations, GSK, Stockley Park, UK;
Yu W, Amgen Inc.; Thousand Oaks, USA
Zetterquist W, Dept. Women's and Children's Health & Centre for Allergy Research, Karolinska Institutet, Stockholm, Sweden
Zolkipli Z, NIHR Southampton Respiratory Biomedical Research Unit, Clinical and Experimental Sciences and Human Development and Health, Southampton, UK;
Zwinderman A H, Academic Medical Centre, University of Amsterdam, The Netherlands;

**Partner organisations**	
Novartis Pharma AG	University of Southampton, Southampton, UK
Academic Medical Centre, University of Amsterdam, Amsterdam, The Netherlands	Imperial College London, London, UK
University of Catania, Catania, Italy	University of Rome 'Tor Vergata', Rome, Italy
Hvidore Hospital, Hvidore, Denmark	Jagiellonian Univ. Medi.College, Krakow, Poland
University Hospital, Inselspital, Bern, Switzerland	Semmelweis University, Budapest, Hungary
University of Manchester, Manchester, UK	Université d'Aix-Marseille, Marseille, France
Fraunhofer Institute, Hannover, Germany	University Hospital, Umea, Sweden
Ghent University, Ghent, Belgium	Ctr. Nat. Recherche Scientifique, Lyon, France
Università Cattolica del Sacro Cuore, Rome, Italy	University Hospital, Copenhagen, Denmark
Karolinska Institutet, Stockholm, Sweden	Nottingham University Hospital, Nottingham, UK
University of Bergen, Bergen, Norway	Netherlands Asthma Foundation, Leusden, NL

European Lung Foundation, Sheffield, UK	Asthma UK, London, UK
European. Fed. of Allergy and Airways Diseases Patients' Associations, Brussels, Belgium	Lega Italiano Anti Fumo, Catania, Italy
International Primary Care Respiratory Group, Aberdeen, Scotland	Philips Research Laboratories, Eindhoven, NL
Synairgen Research Ltd, Southampton, UK	Aerocrine AB, Stockholm, Sweden
BioSci Consulting, Maasmechelen, Belgium	Almirall
AstraZeneca	Boehringer Ingelheim
Chiesi	GlaxoSmithKline
Roche	UCB
Janssen Biologics BV	Amgen NV
Merck Sharp & Dome Corp	

MEMBERS OF THE ETHICS BOARD			
**Name**	**Task**	**Affiliation**	**e-mail**
Jan-Bas Prins	Biomedical research	LUMC/the Netherlands	J.B.Prins@lumc.nl
Martina Gahlemann	Clinical care	BI/Germany	Martina.Gahlemann@boehringer-ingelheim.com
Luigi Visintin	Legal affairs	LIAF/Italy	visintin@inrete.it
Hazel Evans	Paediatric care	Southampton/UK	hazel.evans@uhs.nhs.uk
Martine Puhl	Patient representation (co chair)	NAF/ the Netherlands	martine@puhl.nl
Lina Buzermaniene	Patient representation	EFA/Lithuania	lina.buzermaniene@pavb.lt
Val Hudson	Patient representation	Asthma UK	hudsonval7@gmail.com
Laura Bond	Patient representation	Asthma UK	lvbond22@googlemail.com
Pim de Boer	Patient representation and pathobiology	IND	deboer.pim@hetnet.nl
Guy Widdershoven	Research ethics	VUMC/the Netherlands	g.widdershoven@vumc.nl
Ralf Sigmund	Research methodology and biostatistics	BI/Germany	ralf.sigmund@boehringer-ingelheim.com

THE PATIENT INPUT PLATFORM

Name	Country
Amanda Roberts	UK
David Supple (chair)	UK
Dominique Hamerlijnck	The Netherlands
Jenny Negus	UK
Juliëtte Kamphuis	The Netherlands
Lehanne Sergison	UK
Luigi Visintin	Italy
Pim de Boer (co-chair)	The Netherlands
Susanne Onstein	The Netherlands

MEMBERS OF THE SAFETY MONITORING BOARD	
**Name**	**Task**
William MacNee	Clinical care
Renato Bernardini	Clinical pharmacology
Louis Bont	Paediatric care and infectious diseases
Per-Ake Wecksell	Patient representation
Pim de Boer	Patient representation and pathobiology (chair)
Martina Gahlemann	Patient safety advice and clinical care (co-chair)
Ralf Sigmund	Bio-informatician
